# A challenge in qualitative research: Family members sitting in on interviews about sensitive subjects

**DOI:** 10.1111/hex.13263

**Published:** 2021-05-13

**Authors:** Anna Kathryn Taylor, Steven Armitage, Ambareen Kausar

**Affiliations:** ^1^ Visiting Academic Clinical Fellow in Psychiatry School of Medicine Leeds Institute of Health Sciences Faculty of Medicine and Health University of Leeds Leeds UK; ^2^ Public Contributor UK; ^3^ Consultant HPB Surgeon Department of General Surgery Royal Blackburn Hospital East Lancashire Hospitals NHS Trust Blackburn UK

## CONFLICT OF INTEREST

The authors declare they have no conflicts of interest.

## PATIENT AND PUBLIC CONTRIBUTION

A Patient Advisory Group offered input into the main study design, analysis and dissemination. Steven Armitage is the husband of one of our study participants and offered to co‐produce this paper.

Semi‐structured interviews enable the exploration of a participant's views and experiences, and can reveal in‐depth insights into a problem. Interviews usually consist of a dialogue between the researcher and the study participant.^1^ Dyadic interviewing^2^ is a form of qualitative research in which two participants interact and respond to open‐ended questions posed by the researcher, recognizing and utilizing the interdependent relationship between individuals. This can be a useful approach for some research settings. Researchers may study the relationship between interviewees in addition to the data generated by their interaction, offering a joint perspective on shared problems such as management of chronic illness.^3^ The data from dyadic interviewing can be explored through considering the dyad as a single unit of analysis, or alternatively as two separate perspectives. Individual interviewing involves a single perspective but might enable that participant to speak more openly; there is a risk with dyadic interviewing that a disclosure from one interviewee could cause harm to the other, or one person may dominate the interview.^3^ Therefore, sensitive subjects may be better discussed in individual interviews. Sometimes, researchers encounter family members of study participants who wish to join a one‐to‐one interview; this may create a dilemma.

We have recently completed a qualitative study exploring the psychological impact of recovery following surgery for pancreatic cancer,^4^ which had patient involvement from its inception.^5^

Twenty patients were interviewed. Several participants asked whether family members would take part in these semi‐structured interviews. Some wanted a family member (usually a spouse) to join the interview because they had supported them through the process of diagnosis, surgery and treatment and it was suggested that they may be able to contribute to the discussion. Most, however, preferred to be interviewed unaccompanied. One participant stated that she was relieved to be interviewed without any family members present because she could be more honest about her feelings, and the interview gave her the opportunity to express them freely^4^:

*‘My husband always comes with me to the appointments and I said to him yesterday "where are you going to sit?" because I thought I’m not, I can’t speak openly about things because I don’t want to upset him.’*

​

Our research protocol stipulated that participants would be interviewed alone, and it was therefore crucial that we abided by the protocol that had been approved by the ethics committee. Our patient advisory group had suggested that the interview should be conducted with the person who had had surgery for cancer on their own, to enable them to discuss their concerns without fear of worrying relatives. The interview topic guide focused specifically on the perspectives of the patient, and did not include questions directly exploring family members’ perspectives. In designing our study, we felt that although there were possible benefits to conducting dyadic interviews, there was a risk that the presence of a family member could prevent the participant from disclosing difficult feelings and emotions.^3^ This was borne out by several comments from our participants stating that although they had received support from family and friends, they often had not spoken openly to them about their fears or concerns; it was likely therefore that they would not have disclosed these feelings in a research interview with a family member present.

It is important, however, to recognize that a cancer diagnosis is likely to have a significant psychological impact on family members as well as the individual patient, and there may be a therapeutic value for relatives in engaging in the research process.

We have reflected, throughout our work, that it is possible to balance the needs of the participant with the needs of the family, while also meeting the demands of the research. We were able to achieve this by inviting family members to participate in an informal discussion with the researcher following the interview if they wished. This enabled them to express their own perspectives on the treatment journey of their loved one, as well as allowing them to reflect on their own feelings about their relative's illness, treatment and recovery. Similarly to the principles of dyadic research, the participant and family member were encouraged to reflect together on their experiences. Although we made it clear to our participants and family members that this discussion would not be used as data for the research (and thus we were still working within the remit of our protocol), we were able to utilize it as part of our field notes to provide context for each interview.

Steven Armitage, the husband of one of our study participants, reflected on the benefits of engaging in discussion about his own experiences:

*‘The day she was diagnosed with cancer was undoubtedly the second worst day of my life, the worst being the day of her surgery. Once discharged, rightly so it was her that got all the attention, but it’s very tough coping with your own trauma while supporting and looking after someone going through theirs, having to stay strong while sometimes despairing to almost breaking point. And it’s a long journey through chemo and follow‐up, and there isn’t any support for people like me. Support organizations were unable to help because they had no capacity. Eventually I had to pay privately for therapy. This helped enormously, and I am on antidepressants for anxiety. It can’tbe right that because I had the means to seek help privately I could*.

*Being involved in the study has been for us the opportunity to let people know how hard it can be for the patient as well as their family. It’s been very useful and rewarding to know that this research may help focus people’s minds and ensure that others in our situation in the future may benefit from our experiences.’*

​

Recognizing the depth of impact on family members of people with pancreatic cancer was a by‐product of our study and should be pursued further. We would be interested in the views of other researchers conducting interviews exploring sensitive subjects, around the inclusion of family members in such interviews.

## Data Availability

Data sharing is not applicable to this article as no new data were created or analysed.
